# Supplementary material for: JAK inhibition decreases the autoimmune burden in Down syndrome
Source: medRxiv. 2024 Jun 14:2024.06.13.24308783. Preprint. [Version 1] doi: 10.1101/2024.06.13.24308783 (PMC11213071; doi:10.1101/2024.06.13.24308783)
Supplement: Supplementary file 1 [file NIHPP2024.06.13.24308783v1-supplement-1.pdf]

medRxiv preprint doi: https://doi.org/10.1101/2024.06.13.24308783; this version posted June 14, 2024. The copyright holder for this preprint (which was not certified by peer review) is the author/funder, who has granted medRxiv a license to display the preprint in perpetuity.
It is made available under a CC-BY 4.0 International license .

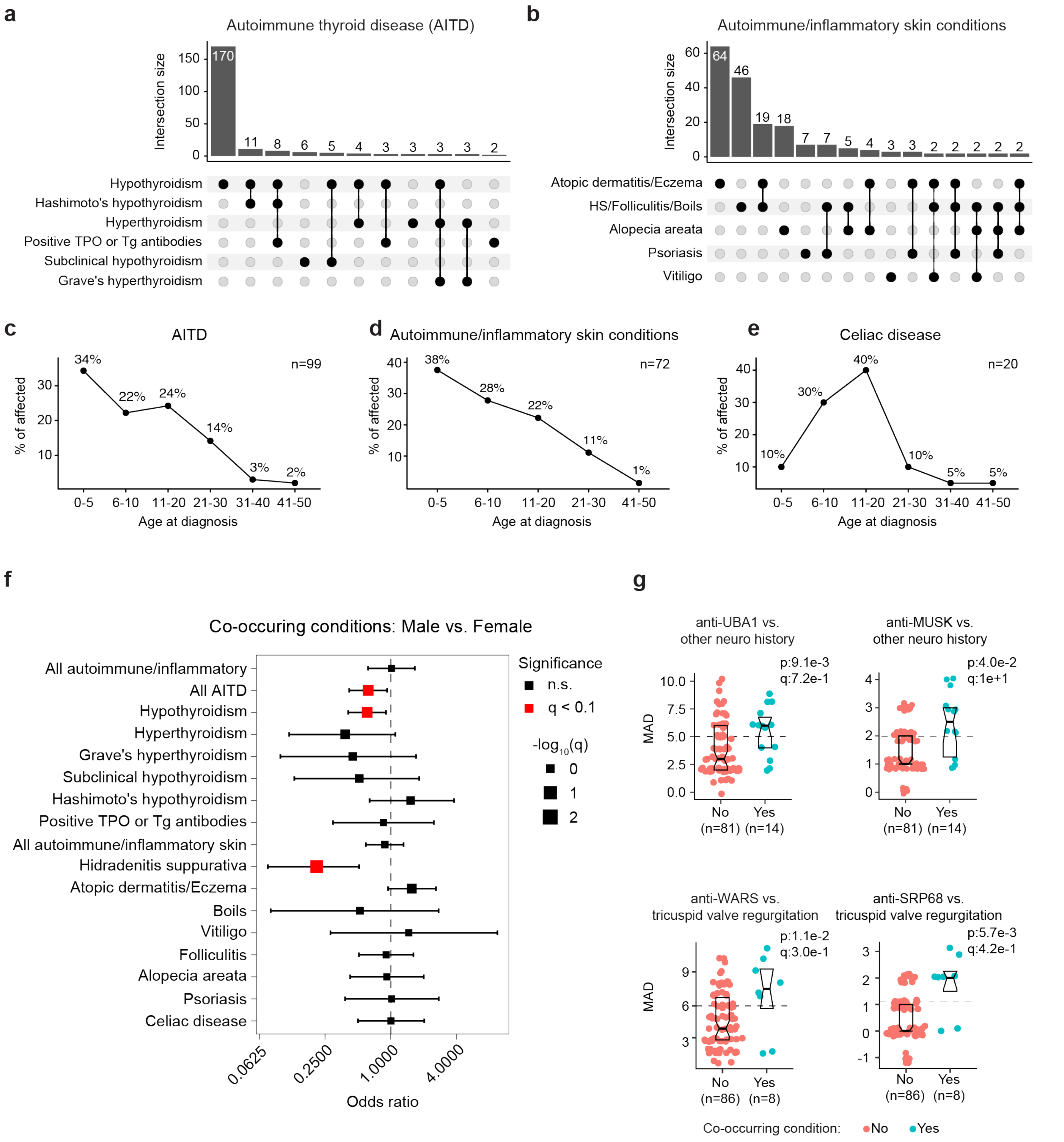

Figure 1 – figure supplement 1. Early onset multi-organ autoimmunity and autoantibody production in Down syndrome. a-b, Upset plots showing overlap between various reported diagnoses indicative of autoimmune thyroid disease (a) or autoimmune/inflammatory skin conditions (b) in research participants with Down syndrome (DS, all ages, n=441) enrolled in the Human Trisome Project (HTP). **c-**

medRxiv preprint doi: https://doi.org/10.1101/2024.06.13.24308783; this version posted June 14, 2024. The copyright holder for this preprint (which was not certified by peer review) is the author/funder, who has granted medRxiv a license to display the preprint in perpetuity.
It is made available under a CC-BY 4.0 International license .

e, Plots showing the percentages of cases by age at diagnosis for AITD (c), autoimmune/inflammatory skin conditions (d), and celiac disease (e). Sample sizes indicated in each chart. **f**, Odds ratio plot for Fisher's exact test of proportions (cases vs. controls in males vs. females) for history of co-occurring conditions in individuals with DS (all ages, total n=441). Conditions with $q < 0.1$ (10% FDR) are highlighted in red. The size of square points is inversely proportional to q value; error bars represent 95% confidence intervals. **g**, Sina plots displaying the levels of select autoantibodies in individuals with DS, with or without history of the indicated co-occurring conditions. MAD: median absolute deviation. Horizontal dashed lines indicate 90th percentiles for the D21 group. Sample sizes are indicated under each plot. q values calculated by Benjamini-Hochberg adjustment of p-values from Fisher's exact tests.

medRxiv preprint doi: https://doi.org/10.1101/2024.06.13.24308783; this version posted June 14, 2024. The copyright holder for this preprint (which was not certified by peer review) is the author/funder, who has granted medRxiv a license to display the preprint in perpetuity.
It is made available under a CC-BY 4.0 International license .

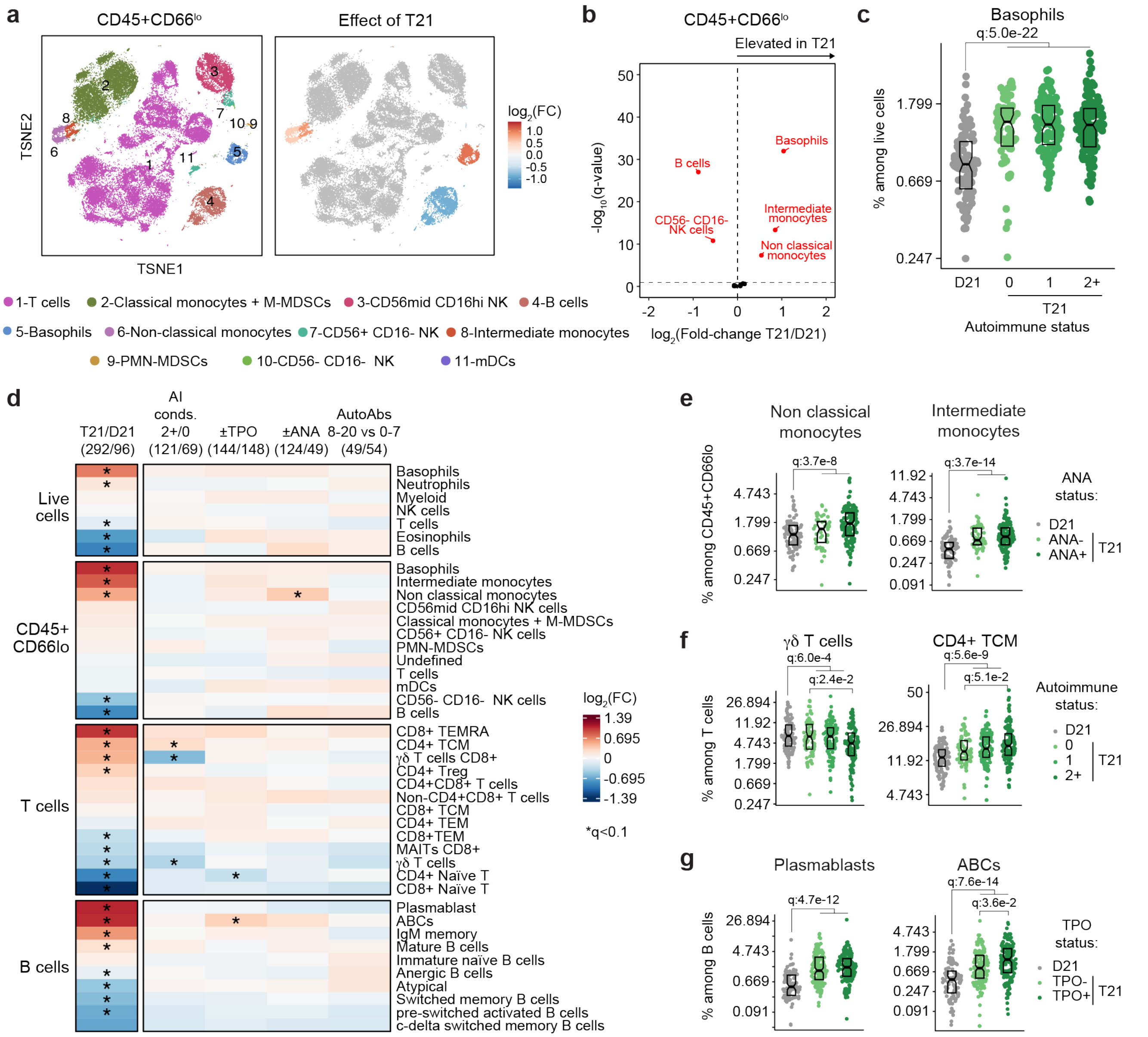

Figure 2 – figure supplement 1. Consistent remodeling of the peripheral immune system in Down syndrome. a, t-distributed Stochastic Neighbor Embedding (t-SNE) plot displaying major immune populations identified by FlowSOM analysis of mass cytometry data for CD45+ CD66^{lo} non-granulocytes (left) and color coded by the impact of trisomy 21 (T21) on their relative frequency (right). Red indicates increased frequency and blue indicates decreased frequency among research participants with T21 (n=292) versus euploid controls (D21, n=96). **b**, Volcano plot showing the results of beta regression analysis of immune cell populations among CD45+ CD66^{lo} non-granulocytes from research participants with T21 (n=292) versus euploid controls (D21, n=96). The dashed horizontal line indicates

medRxiv preprint doi: https://doi.org/10.1101/2024.06.13.24308783; this version posted June 14, 2024. The copyright holder for this preprint (which was not certified by peer review) is the author/funder, who has granted medRxiv a license to display the preprint in perpetuity.
It is made available under a CC-BY 4.0 International license .

a significance threshold of 10% FDR (q<0.1) after Benjamini-Hochberg correction for multiple testing. **c**, Frequencies of basophils among all live cells in euploid controls (D21, n=96) versus individuals with T21 and history of 0 (n=44), 1 (n=71) or 2+ (n=88) autoimmune/inflammatory conditions. Data is displayed as modified sina plots with boxes indicating quartiles. **d**, Heatmap summarizing the results of beta regression testing for differences in frequencies of indicated immune cell populations among all live cells, CD45+ $CD66^{lo}$ non-granulocytes, T cells, and B cells by T21 (n=292) versus D21 (n=96) status, or by different subgroups within the T21 cohort: 2+ (n=88) versus 0 (n=44) autoimmune/inflammatory conditions; TPO+ (n=144) versus TPO- (n=148); ANA+ (n=124) versus ANA- (n=49); or positivity for 8-20 (n=49) versus 0-7 (n=54) autoantibodies elevated in DS. Asterisks indicate significance after Benjamini-Hochberg correction for multiple testing (q<0.1, 10% FDR). **e-g**, Representative examples of immune cell populations from d, showing effects of ANA positivity (e), number of autoimmune conditions (f), and TPO status (g). Data are presented as modified sina plots with boxes indicating quartiles, with q-values indicating beta regression significance after Benjamini-Hochberg correction for multiple testing.

medRxiv preprint doi: https://doi.org/10.1101/2024.06.13.24308783; this version posted June 14, 2024. The copyright holder for this preprint (which was not certified by peer review) is the author/funder, who has granted medRxiv a license to display the preprint in perpetuity.
It is made available under a CC-BY 4.0 International license .

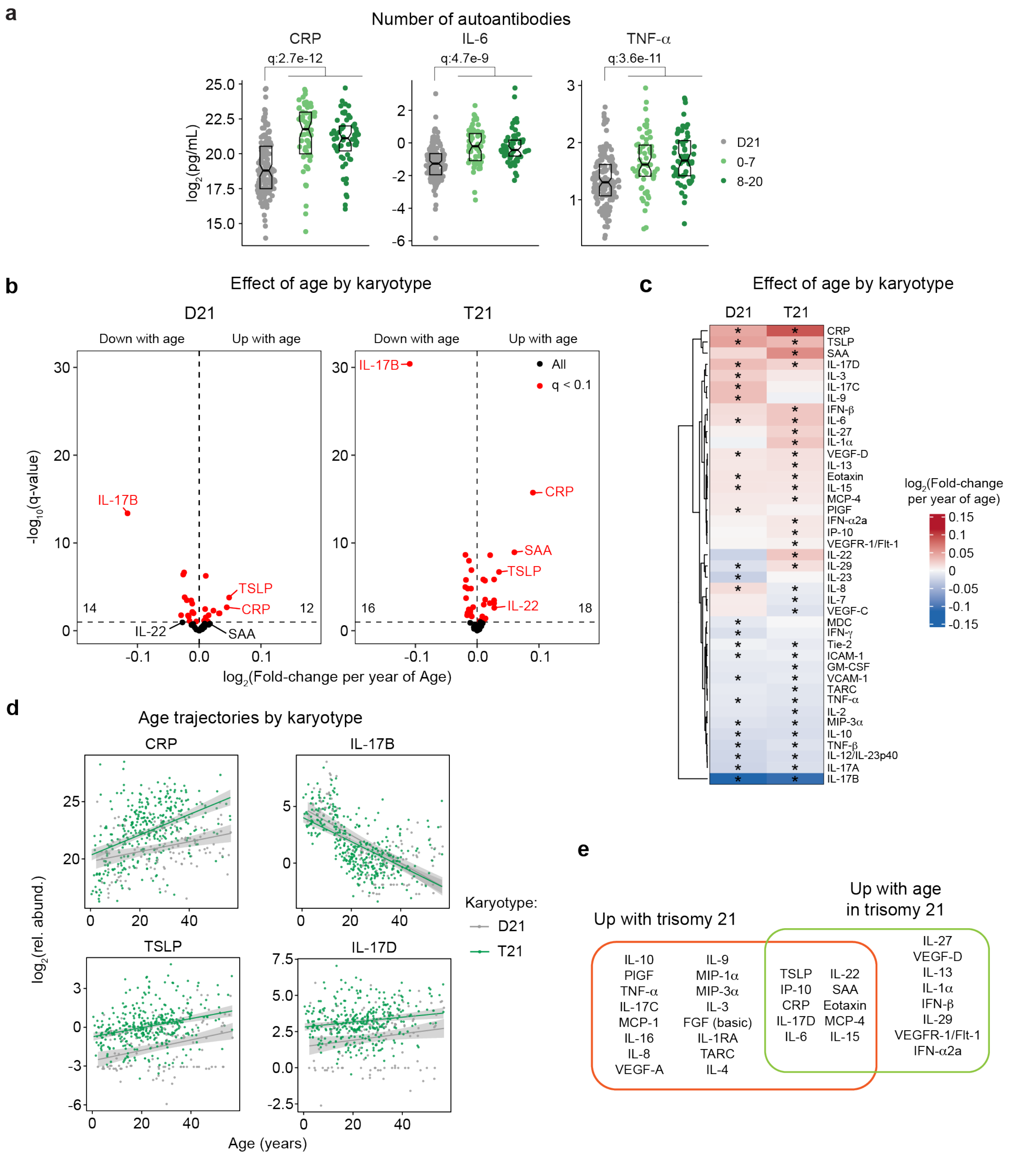

Figure 3 – figure supplement 1. Consistent hypercytokinemia from an early age in Down syndrome. a, Comparison of CRP, IL-6, and TNF-α levels in euploid controls (D21, n=131) versus subsets of individuals with T21 based on number of autoantibodies commonly elevated in Down syndrome: 0-7 autoantibodies (n=62) versus 8-20 autoantibodies (n=57). Data are presented as modified

medRxiv preprint doi: https://doi.org/10.1101/2024.06.13.24308783; this version posted June 14, 2024. The copyright holder for this preprint (which was not certified by peer review) is the author/funder, who has granted medRxiv a license to display the preprint in perpetuity.
It is made available under a CC-BY 4.0 International license .

sina plots with boxes indicating quartiles. q-values indicate linear regression significance after Benjamini-Hochberg correction for multiple testing. **b**, Volcano plots presenting the results of linear regression testing for association between age and the levels of 54 immune markers in the plasma of euploid controls (left, D21, n=131) and individuals with trisomy 21 (right, T21, n=346) enrolled in the Human Trisome Project (HTP) study. Horizontal dashed lines indicate a significance threshold of 10% FDR ($q<0.1$) after Benjamini-Hochberg correction for multiple testing. **c**, Heatmap comparing the effect of age on levels of immune markers in D21 and T21. Heatmap color scale represents log2-transformed mean fold-change per year of age; asterisks indicate significance ($q < 0.1$) for linear regression testing. **d**, Scatter plots showing the age trajectories of select immune markers in D21 versus T21. Sample sizes as in c. Lines represent least squares linear fits with shaded areas indicating 95% confidence interval. **e**, Diagram representing the overlap between immune markers elevated in T21 versus D21 and those elevated with age in T21.

medRxiv preprint doi: https://doi.org/10.1101/2024.06.13.24308783; this version posted June 14, 2024. The copyright holder for this preprint (which was not certified by peer review) is the author/funder, who has granted medRxiv a license to display the preprint in perpetuity.
It is made available under a CC-BY 4.0 International license .

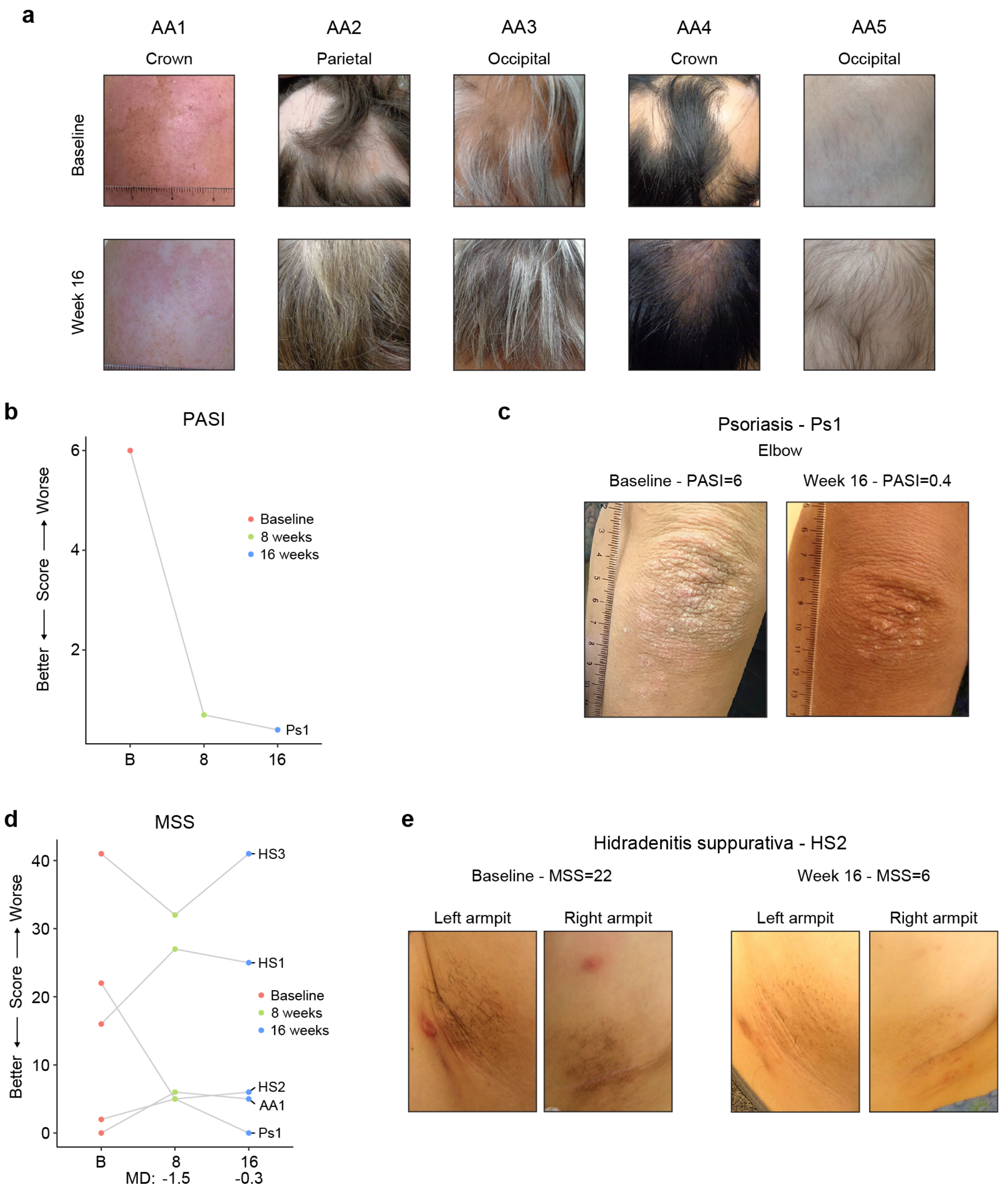

Figure 5 – figure supplement 1. Tofacitinib improves diverse skin pathologies in Down syndrome.

a, Images of five participants with alopecia areata at baseline and after 16 weeks of tofacitinib treatment. **b-c**, Psoriasis Area and Severity Index score (b) and images (c) for participant with psoriatic arthritis. **d,** Modified Sartorius Scale (MSS) scores for five participants with hidradenitis suppurativa (HS). MD:

medRxiv preprint doi: https://doi.org/10.1101/2024.06.13.24308783; this version posted June 14, 2024. The copyright holder for this preprint (which was not certified by peer review) is the author/funder, who has granted medRxiv a license to display the preprint in perpetuity.
It is made available under a CC-BY 4.0 International license .

median difference. **e**, Images for participant affected by HS at baseline and 16-week endpoint visit. p values not shown as per interim analysis plan.

medRxiv preprint doi: https://doi.org/10.1101/2024.06.13.24308783; this version posted June 14, 2024. The copyright holder for this preprint (which was not certified by peer review) is the author/funder, who has granted medRxiv a license to display the preprint in perpetuity.
It is made available under a CC-BY 4.0 International license .

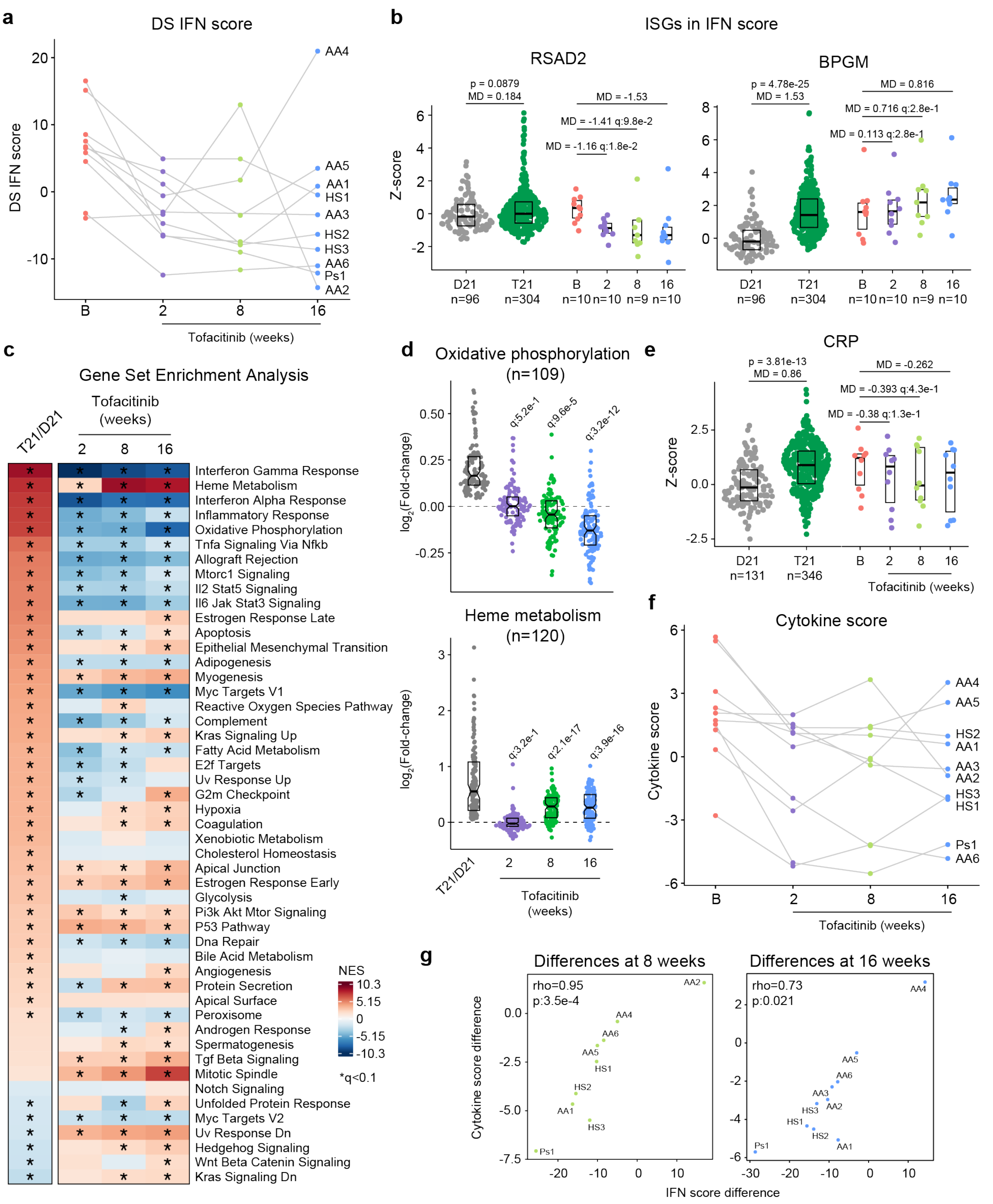

Figure 6 – figure supplement 1. JAK inhibition reduces multiple markers of inflammation and autoimmunity in Down syndrome. a, Plot showing trajectory of IFN scores derived from whole blood transcriptome for 10 clinical trial participants at baseline (B), versus 2, 8 and 16 weeks of tofacitinib

medRxiv preprint doi: https://doi.org/10.1101/2024.06.13.24308783; this version posted June 14, 2024. The copyright holder for this preprint (which was not certified by peer review) is the author/funder, who has granted medRxiv a license to display the preprint in perpetuity.
It is made available under a CC-BY 4.0 International license .

treatment. **b**, Comparison of ISG expression in the whole blood transcriptome data from research participants in the Human Trisome Project (HTP) cohort study by karyotype status (D21, grey; T21, green) and the clinical trial cohort at baseline (B), and weeks 2, 8 and 16 of tofacitinib treatment. Data are represented as modified sina plots with boxes indicating quartiles. Sample sizes are indicated below x-axis. Horizontal bars indicate comparisons between groups with median differences (MD) with p-values from Mann-Whitney U-tests (HTP cohort) and q-values from paired Wilcox tests (clinical trial). **c,** Heatmap displaying the results of Gene Set Enrichment Analysis (GSEA) of global transcriptome changes in the whole blood RNA of research participants in the HTP cohort (T21, n=304; D21, n=96) versus the clinical trial cohort at 2 (n=10), 8 (n=9), and 16 weeks (n=10) of tofacitinib treatment relative to baseline (n=10). Asterisks indicate significance after correction by Benjamini-Hochberg method for multiple testing (q<0.1, 10% FDR). NES: normalized enrichment score. **d,** Analysis of fold changes for 109 genes involved in oxidative phosphorylation and 120 genes involved in heme metabolism significantly elevated in Down syndrome (T21 versus D21 in the HTP cohort) versus the clinical trial cohort at 2, 8 and 16 weeks of tofacitinib treatment relative to baseline. Sample numbers as in c. **e,** Comparison of CRP levels in the HTP cohort by karyotype status (D21, grey; T21, green) versus the clinical trial cohort at baseline (B) and 2, 8 and 16 weeks of tofacitinib treatment. Data are represented as modified sina plots with boxes indicating quartiles. Sample sizes are indicated below x-axis. Horizontal bars indicate comparisons between groups with median differences (MD) with p-values from Mann-Whitney U-tests (HTP cohort) and q-values from paired Wilcox tests (clinical trial). **f,** Plot showing trajectory of cytokine scores for 10 clinical trial participants at baseline (B), versus 2, 8 and 16 weeks of tofacitinib treatment. **g,** Plots showing Spearman correlations between fold changes in IFN scores versus cytokine scores at 8 and 16 weeks of tofacitinib treatment versus baseline. Sample size is n=10.

medRxiv preprint doi: https://doi.org/10.1101/2024.06.13.24308783; this version posted June 14, 2024. The copyright holder for this preprint (which was not certified by peer review) is the author/funder, who has granted medRxiv a license to display the preprint in perpetuity.
It is made available under a CC-BY 4.0 International license .

Supplementary File Legends.

Supplementary file 1. (A) Cohort characteristics and (B) autoimmune conditions for Human Trisome Project participants analyzed in this study.

Supplementary file 2. Autoantibody measurements of Human Tisome Project participants: (A) anti-thyroid peroxidase (TPO) reactivity; (B) anti-nuclear antigen (ANA) reactivity; (C) SciLifeLabs autoantigen peptide array data.

Supplementary file 3. (A) Minimum qualifying scores for skin conditions. (B) Cohort characteristics for clinical trial participants.

Supplementary file 4. Adverse events for clinical trial participants.

Supplementary file 5. Skin pathology metrics for clinical trial participants: (A) Investigator's Global Assessment (IGA); (B) Dermatology Life Quality Index (DLQI); (C) Severity of Alopecia Tool (SALT); (D) Psoriasis Area and Severity Index (PASI); and (E) Eczema Area and Severity Index (EASI).

Supplementary file 6. (A) DS IFN scores; (B) Cytokine scores; (C) anti-thyroid peroxidase (TPO) titers; (D) anti-transglutaminase (TG) titers; and (E) anti-thyroid stimulating hormone receptor (TSHR) titers for clinical trial participants.

Supplementary file 7. Marker information for mass cytometry analysis.

Supplementary file 8. Clinical trial protocol.
